# The statistics of identifying differentially expressed genes in Expresso and TM4: a comparison

**DOI:** 10.1186/1471-2105-7-215

**Published:** 2006-04-20

**Authors:** Allan A Sioson, Shrinivasrao P Mane, Pinghua Li, Wei Sha, Lenwood S Heath, Hans J Bohnert, Ruth Grene

**Affiliations:** 1 Department of Computer Science, Virginia Tech, Blacksburg, USA; 2 Department of Plant Pathology, Physiology and Weed Science, Virginia Tech, Blacksburg, USA; 3 Department of Plant Biology and Department of Crop Sciences, University of Illinois, Urbana, USA; 4 Virginia Bioinformatics Institute, Virginia Tech, Blacksburg, USA

## Abstract

**Background:**

Analysis of DNA microarray data takes as input spot intensity measurements from scanner software and returns differential expression of genes between two conditions, together with a statistical significance assessment. This process typically consists of two steps: data normalization and identification of differentially expressed genes through statistical analysis. The Expresso microarray experiment management system implements these steps with a two-stage, log-linear ANOVA mixed model technique, tailored to individual experimental designs. The complement of tools in TM4, on the other hand, is based on a number of preset design choices that limit its flexibility. In the TM4 microarray analysis suite, normalization, filter, and analysis methods form an analysis pipeline. TM4 computes integrated intensity values (IIV) from the average intensities and spot pixel counts returned by the scanner software as input to its normalization steps. By contrast, Expresso can use either IIV data or median intensity values (MIV). Here, we compare Expresso and TM4 analysis of two experiments and assess the results against qRT-PCR data.

**Results:**

The Expresso analysis using MIV data consistently identifies more genes as differentially expressed, when compared to Expresso analysis with IIV data. The typical TM4 normalization and filtering pipeline corrects systematic intensity-specific bias on a per microarray basis. Subsequent statistical analysis with Expresso or a TM4 *t*-test can effectively identify differentially expressed genes. The best agreement with qRT-PCR data is obtained through the use of Expresso analysis and MIV data.

**Conclusion:**

The results of this research are of practical value to biologists who analyze microarray data sets. The TM4 normalization and filtering pipeline corrects microarray-specific systematic bias and complements the normalization stage in Expresso analysis. The results of Expresso using MIV data have the best agreement with qRT-PCR results. In one experiment, MIV is a better choice than IIV as input to data normalization and statistical analysis methods, as it yields as greater number of statistically significant differentially expressed genes; TM4 does not support the choice of MIV input data. Overall, the more flexible and extensive statistical models of Expresso achieve more accurate analytical results, when judged by the yardstick of qRT-PCR data, in the context of an experimental design of modest complexity.

## Background

DNA microarrays are a powerful means of monitoring the expression of thousands of genes simultaneously. A variety of computational and statistical methods have been proposed to extract information from the large quantity of data generated from microarray experiments. Many methods assume, as we do here, the use of cDNA labeled with one of two fluorescent dyes to differentiate two treatments on a single microarray, implying data from two images to be analyzed. These methods include a number of data normalization techniques to reduce the effects of systematic errors and various kinds of statistical tests to identify differentially expressed genes in comparisons among different experimental conditions. There is as yet no single method that can be recommended under all circumstances for either normalization or identification of differential gene expression.

In recent years, ANOVA methods have gained popularity for identification of differential gene expression. The power of ANOVA methods derives from their flexibility in fitting and comparing different models to a given set of data [1]. One such method is the two-stage, log-linear ANOVA mixed models technique of Wolfinger, *et al*., [2]. Its first stage uses a normalization model designed to remove global effects across all microarrays. Its second stage uses a gene-specific model to estimate gene-treatment interactions as ratios of gene expression under control and treated conditions, along with a statistical significance. Kerr [3] notes that the global normalization model employed in this technique is conducive to combining data across genes for realistic and robust models of error, especially when random effects are included. Pan [4] compares different microarray statistical analysis methods and demonstrates that the log-linear ANOVA mixed model approach performs better than the *t*-test and regression approaches. The regression approach, although flexible and robust, assumes that the data is drawn from a normal distribution, while the *t*-test is limited due to very few degrees of freedom. Chu, *et al*., [5] compare two log-linear ANOVA mixed models for probe-level, oligonucleotide array data and found that both types of models capture key measurable sources of variability of oligonucleotide arrays for real and simulated data. Cui and Churchill [6] review the use of a mixed ANOVA model for analyzing a cDNA microarray experiment and conclude that such models provide a powerful way to obtain information from experiments with multiple factors or sources of variation. Rosa, *et al*., [7] review issues of analyzing cDNA microarrays with mixed linear models and puts such analysis in the larger context of Bayesian analysis procedures and adjustments for multiple testing.

Data normalization is the first step in analyzing microarray data; numerous data normalization methods have been proposed and investigated. While refinements of existing methods continue to appear (e.g., Futschik and Crompton [8]), naive methods, such as total intensity normalization, are still in use (e.g., Held, *et al*., [9]). Xie, *et al*., [10] did a comparative study of normalization methods and test statistics to analyze the results of a DNA-protein binding microarray experiment. Using performance and bias correction criteria, Bolstad, *et al*., [11] evaluate the cyclic lowess method, the contrast method, the quantile method, and baseline array scaling methods, both linear and non-linear; they demonstrate that normalization methods incorporating data from all microarrays perform better than methods employing a baseline array.

Several software tools that combine data normalization and statistical analysis are currently available. Dudoit, *et al*., [12] review these software tools with an emphasis on the TM4 microarray software suite, Bioconductor in R, and the BioArray Software Environment (BASE) system. Saeed, et al., [13] describe the features and capabilities of TM4, while Quackenbush [14] describes the normalization and transformation methods implemented in it. Williams, et al., [15], Zhu, et al., [16], and Khaitovich, et al., [17] have used TM4 in microarray data analysis. Another system is Expresso, an experiment management system that serves as a unifying framework to study data driven applications such as microarray experiments [18-20]. Expresso has adapted the two-stage ANOVA mixed models technique of Wolfinger, et al., [2] to the particular needs of individual microarray data sets. Our experience with numerous such data sets has demonstrated that modeling the underlying experiment carefully and completely is essential to obtaining meaningful and defensible results. Use of tools that require experiments to conform to their analysis methods are less than satisfactory.

In this paper, we compare the Expresso analysis methodology to the approach provided in the TM4 microarray analysis software suite [13]. Each is invoked to identify differentially expressed genes in two experimental data sets, each of which uses an *Arabidopsis thaliana *oligonucleotide array. Along the way, we demonstrate differences between the use of integrated intensity values (IIV) and median intensity values (MIV) as inputs. We report interactions between normalization and gene identification methods. We use quantitative reverse-transcriptase PCR (qRT-PCR) results to assess the consistency of genes reported by TM4 and Expresso as having significant differential expression.

## Results and discussion

**Figure 1:**
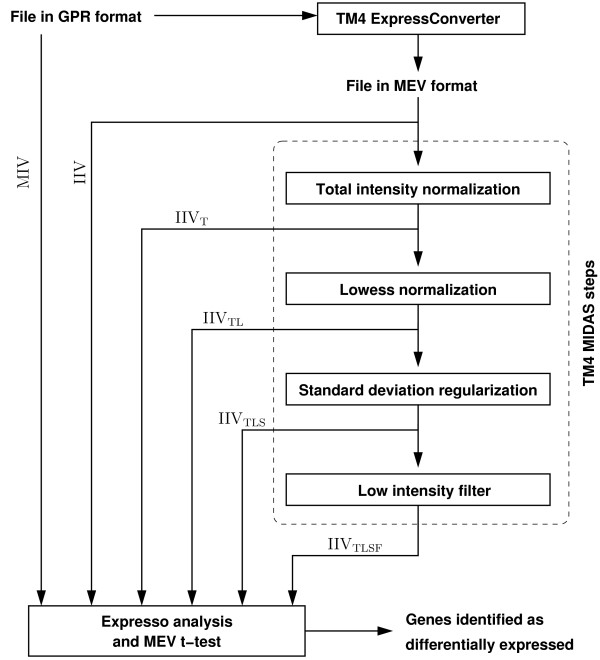
**Overall flow of the statistical analyses of microarray data**. Data input is in GPR format and provides the MIV for each spot. TM4 analysis requires ExpressConverter to generate MEV format containing the IIV for each spot. The normalization steps performed by MIDAS are T (total intensity normalization), L (lowess normalization), S (standard deviation regularization), and F (low intensity filter). Differential gene expression is obtained from the Expresso GP model, the Expresso GOT model (Experiment 2 only), and the *t*-test in MEV.

Here, we report a portion of the results obtained in our comparison of Expresso analysis and the TM4 pipeline (see Materials and Methods). Figure 1 illustrates the overall flow of the statistical analyses of microarray data that were done in this study. We began with microarray data in GPR format from Experiment 1 and Experiment 2. Median intensity values (MIV) from the GPR files can be analyzed by the Expresso GP and GOT models directly. ExpressConverter provides integrated intensity values (IIV) for further Expresso and TM4 analysis. The MIDAS normalization and filtering pipeline executes these steps in order: total intensity normalization (subscript T), lowess normalization (subscript L), standard deviation regularization (subscript S), and low intensity filter (subscript F). MIDAS allows tapping the output of any step in the pipeline; for example, IIV_TL _signifies an MEV file after total intensity normalization followed by lowess normalization. The identification of genes with significant differential expression was performed on all GPR and MEV files, using the Expresso GP and GOT models and the *t*-test in MEV.

### Normalization and low intensity filtering in TM4

Quackenbush [14] describes the use of ratio-intensity plots (RI-plots) to detect and normalize for any systematic intensity-dependent dye bias using lowess normalization (see Materials and Methods). We evaluated the effect of lowess normalization within the context of the flow in Figure 1 by creating RI-plots after each step for the second replicate microarray in Experiment 1, WT plant. Supplementary Figure 1 - see Additional file: 1 contains these RI-plots. The IIV_TL _is indeed effective, for this data set, in correcting systematic dye bias, suggesting that preprocessing by these two normalization steps in MIDAS may be a good practice in many situations.

The normalization and filtering pipeline affects the number of genes identified as differentially expressed in both the GP and GOT models. See Table 1. For example, in Experiment 1, the GP model using IIV input data identifies 567 up-expressed genes in the WT microarrays, while it identifies only 460 WT genes as up-expressed if IIV_TLSF _(processed by the complete MIDAS pipeline) input data is used.

Small changes in the number of genes identified as up- or down-expressed after successive MIDAS steps may mask larger changes in the composition of sets of up- and down-expressed genes. To obtain a more precise view of the effects of MIDAS changes, we computed retention counts (RC) and retention percentages (RP) between the gene successive sets whose numbers are in Table 1. RC is the number of genes in the set before the MIDAS step that remain in the set after the step. RP is the percentage of remaining genes with respect to the number of genes in the set after the MIDAS step. Table 2 contains the RC and RP values corresponding to the counts in Table 1. For Experiment 1, there is a tremendous drop in retention during the lowess normalization that follows the total intensity normalization. There is not a drop of corresponding magnitude for Experiment 2. For both experiments, normalization has a significant effect on the sets of genes identified as differentially expressed.

In Experiment 1 results, the number of genes commonly assessed by Expresso as significantly expressed when using IIV and IIV_T _is high. For example, there is 95.45% retention of WT genes (545 total) assessed as up-expressed when using IIV_T _data in Expresso compared to that when using IIV data. Retention percentage of these genes assessed as expressed however went down after doing lowess normalization. There is only 15.20% retention of WT genes (74 total) assessed as up-expressed in the results when using IIV_TS _data in Expresso compared to when using IIV_T_. While we observe increase in the retention percentage in IIV_TLS _(from IIV_TL_) and IIV_TLSF _(from IIV_TLS_), there's low retention percentage in the results using IIV_TLSF _from IIV data. This can be traced in the low retention percentage of IIV_TL _from IIV_T_. Hence, the normalization method that affects the results in Experiment 1 the most is lowess normalization.

The results of Expresso on Experiment 2 show that low retention percentages happen after appli-cation of total intensity normalization (lowest is 59.30%) and after application of low intensity filtering (lowest is 61.19%). The low retention percentages shown in the IIV ∩ IIV_TLSF _column implies that the normalization pipeline also significantly affects the results in Expresso analysis of Experiment 2.

### Choice of intensity signal data

The input to statistical analysis of microarray experiments is a set of real numbers that represent the measured intensity signal for each spot in a microarray. Much statistical analysis of microarray data has traditionally used median intensity values (MIV). The alternative used in TM4 is the integrated intensity value (IIV). (See Materials and Methods.) Since IIV is intended to integrate the measured intensity across the biological sample printed at a spot, one might expect IIV to be a more accurate assessment of the biological measurement than MIV data. For example, a spot having 100 pixels and a median intensity of 5,000 has the same IIV as a spot having 50 pixels and a median intensity of 10,000.

This study provides the opportunity to observe the difference that choosing MIV or IIV makes on the sets of genes ultimately identified as differentially expressed. We used the GP model to analyze unnormalized MIV and IIV data from Experiment 1, and we used the GOT model to analyze unnormalized MIV and IIV data from Experiment 2. Table 3 reports a summary of the results. In Experiment 1, 725 WT genes are assessed as up-expressed and 774 WT genes are down-expressed when MIV data are used in Expresso. These numbers decreased to 567 up-expressed genes and 552 down-expressed genes when IIV data are used instead. A similar trend is observed in Experiment 2 results when using MIV and IIV data. These results suggest that employing IIV input data with Expresso analysis leads to more conservative results than employing MIV input data.

### Comparison of statistical methods

We compared the performance of the GP model, the GOT model, and the *t*-test of MEV in identifying differentially expressed genes in Experiment 2. We used the IIV_TLSF _data of Experiment 2 as input to these methods. We also contrast these results with MIV data analyzed in GP. Table 4 reports counts for these analyses.

**Figure 2:**
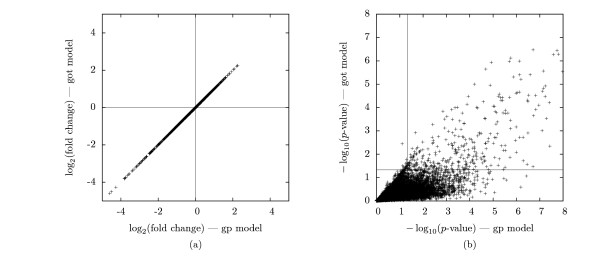
**Comparison of GP and GOT models**. Comparison of estimated log_2_(fold change) and the corresponding *p*-value estimates for the GP and GOT model results of the WS ecotype values in Table 4 (a) This is a scatter plot of the estimated log_2_(fold change) values from the GP and GOT models; these values are essentially identical, (b) This is a scatter plot of the - log_10 _(p-value), again for the GP and GOT models. The dotted lines correspond to - log_10_(0.05), as our significance cutoff is 0.05. For the GP model, it is the points to the right of the dotted line that are significant. For the GOT model, it is the points above the dotted line that are significant.

The plot in Figure 2a demonstrates that the estimates of Iog_2_(fold change) are the same in GP and GOT. As Figure 2b shows, the *p *values by GOT are smaller compared to the *p *values calculated by GP. Use of the MEV *t*-test resulted in fewer genes assessed as significantly expressed when compared to the numbers for the GP model. The results obtained when MIV data was used as input to GP, is closest to the results when using IIV_TLSF_.

To compare the effectiveness of Expresso and TM4 in identifying gene differential expression, we compared the identified direction of differential expression of a select set of genes per genotype in Experiment 2 with results obtained by qRT-PCR. See Table 5. The lowest overall percentage (71.9%) of agreement is between the qRT-PCR results and the MEV *t*-test results using IIV_TLSF_. The log(fold change) estimates of the GP model has 77.1% percentage agreement with the qRT-PCR results, which is slightly higher than the percentage for the MEV *t*-test. The results of the GP model using MIV data demonstrated the greatest agreement, 90.1%, with the qRT-PCR results.

**Figure 3:**
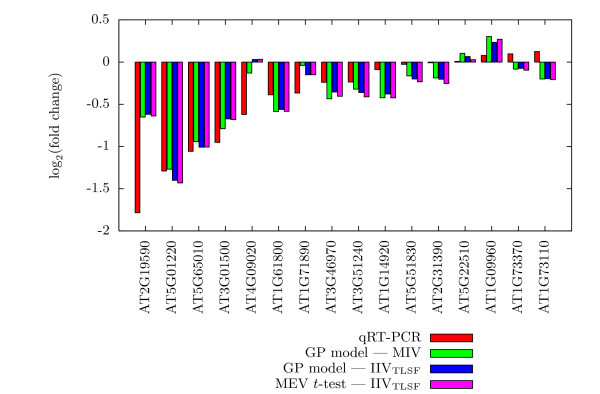
**Comparison of qRT-PCR results, Part I**. Comparison of qRT-PCR results to Expresso and MEV *t*-test results for first 16 of 50 selected genes of the Col-0 genotype of Experiment 2.

**Figure 4:**
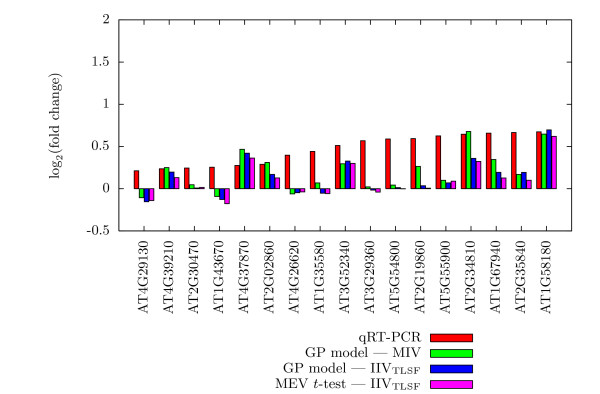
**Comparison of qRT-PCR results, Part II**. Comparison of qRT-PCR results to Expresso and MEV *t*-test results for the middle 17 of 50 selected genes of the Col-0 genotype of Experiment 2.

**Figure 5:**
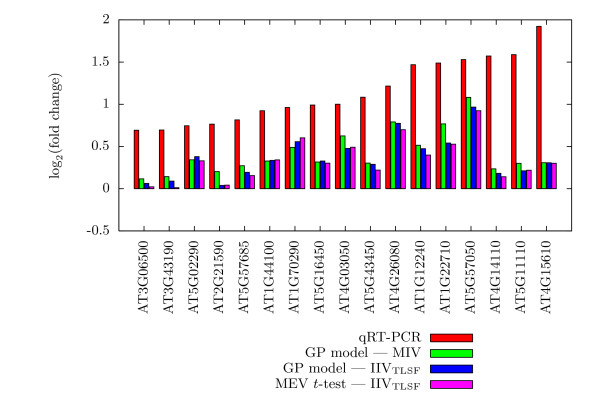
**Comparison of qRT-PCR results, Part III**. Comparison of qRT-PCR results to Expresso and MEV *t*-test results for the last 17 of 50 selected genes of the Col-0 genotype of Experiment 2.

Figures 3, 4, and 5 present the actual assessed Iog_2_(fold change) values for 50 selected Col-0 genes in Experiment 2, along with their qRT-PCR values. These are the 50 Col-0 genes, among the 55 with qRT-PCR values, for which we have expression values for all methods. For each gene, a histogram of the Iog_2_(fold change) estimates of qRT-PCR, the GP model using MIV, the GP model using IIV_TLSF_, and the MEV *t*-test is given. The 50 histograms are spread over three figures to enhance readability and are in increasing order by qRT-PCR estimated change. In general, the log_2_(fold change) estimates of the GP model and of the MEV *t*-test, all IIV_TLSF _input data, are approximately the same, while being slightly different from estimates of the GP model using MIV input data. As might be expected, disagreement between qRT-PCR and microarray results are more prevalent for small estimated log_2_(fold change) values. The histograms for genes AT4G09020 (Figure 3), AT1G35580 (Figure 4), and AT3G29360 (Figure 4) show that the direction of log(fold change) estimate of qRT-PCR matches the direction of the GP model using MIV input data, while differing from the direction of the estimates of the GP model and the MEV *t*-test using IIV_TLSF _input data.

Table 6 summarizes genotype-specific correlation results, which demonstrate that the GP model us-ing MIV input data has the highest correlation with qRT-PCR compared to the GP model and the MEV *t*-test using IIV_TLSF _input data. The highest correlation of 0.85 is for the Col-0 results of qRT-PCR versus the GP model using MIV input. The corresponding correlations for Cvi-0, WS, and Th are 0.73, 0.66, and 0.84, respectively, which are all best among the analysis methods.

## Conclusion

Our integration and comparison of Expresso analysis and the capabilities of TM4 has highlighted successes in microarray analysis, some similarities, and some differences. The success of microarray analysis is demonstrated by considerable agreement between qRT-PCR results and the results of all the examined microarray analysis methods. The greatest agreement was found when median intensity value (MIV) inputs were analyzed with the Expresso GP analysis model. We also found that the use of integrated intensity value (IIV) inputs for Expresso analysis consistently resulted in fewer genes identified as differentially expressed when compared to results from MIV inputs. This suggests that the use of IIV inputs is more conservative than the use of MIV inputs, while MIV inputs may give greater agreement to qRT-PCR results than IIV inputs.

Our results demonstrate that the MIDAS normalization and filtering pipeline corrects systematic intensity-dependent dye bias on a per microarray basis. The normalization stage in Expresso analysis removes global effects across all microarrays and complements the per microarray normalization methods of MIDAS. The generally better agreement of Expresso analysis with qRT-PCR results when compared to the MEV *t*-test suggests that it would be desirable for MEV to have an ANOVA test that has the greater flexibility of the Expresso gene model.

## Methods

### Median and integrated intensity values

This research considers two ways of measuring spot intensity, one or both of which are reported by typical microarray image processing software. The median intensity value (MIV) of a spot is the median value of all the pixels identified as part of the spot. The integrated intensity value (IIV) of a spot is the total value of all the pixels identified as part of the spot. In this research, both are background-corrected values. If the IIV data is unavailable, but the radius of the bounding circle of the spot and its average intensity value are available, then the IIV data can be estimated as the product of the average intensity and the number of pixels in the circle. This is the estimate used by ExpressConverter (below) when it converts GPR format data to MEV format data.

### Microarray data sets

We used data sets from two experiments that used the Arabidopsis Oligonucleotide Microarrays [21], which include 25,712 elements, each a gene-specific 70-mer (Qiagen/Operon, Valencia, CA) for a known or putative open reading frames in *Arabidopsis thaliana*. There are 48 blocks per microarray, 25 rows by 24 columns (600 spots) per block, and 28,800 spots per microarray, including spots for the 25,712 gene-specific 70-mers and 302 control elements. The remaining 2,786 spots are blank.

#### Experiment 1

Experiment 1 compares the responses of *Arabidopsis thaliana *wild type, ecotype Columbia (henceforth, WT), and of an antisense plant for phospholipase D α (antiPLD) in Columbia background to drought stress [22]. Plants were harvested at a single time point, and two biological replicate hybridizations were done for each of WT and antiPLD. Scan Array Express (PerkinElmer Life and Analytical Sciences, Inc., Boston, MA USA) was used to quantitate the four microarrays. By default, ScanArray Express performs a global lowess normalization of median intensities per microarray. ScanArray Express output its results to four files in GenePix (GPR) format, which constitute the Experiment 1 data set.

#### Experiment 2

Li, et al., [23] compare the responses to elevated CO_2 _of a wild *Arabidopsis thaliana *relative (*Thellungiella halophila*, ecotype Shandong; Th) and of three *Arabidopsis thaliana *ecotypes: Wassilewskija (WS), Columbia (Col-0), and Cape Verde Islands (Cvi-0). Three biological replicate hybridizations were done for each genotype. GenePix (Axon Instruments, Union City, CA USA) was used to quantitate the twelve microarrays. GenePix also performs by default a global lowess normalization of median intensities per microarray. The output of GenePix is twelve GPR files, which constitute the Experiment 2 data set.

### Real-time quantitative RT-PCR

For verification of microarray results in Experiment 2, Li, et al., [23] performed real-time quantitative reverse-transcriptase PCR (qRT-PCR) for selected genes — 55 in Col-0; 52 in Cvi-0; 59 in WS; 26 in Th. Supplementary Table 1 - see Additional file: 2 contains the annotation of the selected genes. In brief, primer pairs were selected to represent unique sequences in the *Arabidopsis thaliana *genome and in the *Thellungiella *sequences deposited in NCBI. *Thellungiella *actin (CX129618) cDNA primers and *Arabidopsis thaliana*. Ubiquitin-10 cDNA primers were used as internal controls in the qRT-PCR analyses. RT-PCR products were detected using the fluorescent dye SYBR-green (Applied Biosystems, Foster City, CA USA) and the ABI PRISM/Taqman 7900 Sequence Detection System (Applied Biosystems, Foster City, CA USA). Dissociation curves were generated for each reaction to ensure specific amplification. Three repeats were done for each gene. The averaged threshold cycle numbers were used to estimate original mRNA levels.

The microarray results of Experiment 2 suggested that exposure to elevated CO2 resulted in changes in expression of many genes associated with carbon metabolism, and those associated with pho-tosynthetic carbon metabolism in particular. This included genes encoding proteins that transport pho-tosynthate out of the chloroplast, where carbon fixation takes place, for export to other parts of the cell, and also genes encoding transport proteins that export carbon skeletons out of the cell to other tissues where growth is taking place. Because of this finding, it was important to validate the results obtained for gene expression associated with carbon metabolism with qRT-PCR. Hence, a number of the genes in Supplementary Table 1 - see Additional file: 2 are related to carbon metabolism.

### Expresso analysis

Expresso analysis employs a general and flexible method to identify differentially expressed genes that is adapted from the two-stage analysis method of Wolfinger, et al., [2]. In general, Expresso analysis consists of two log-linear ANOVA mixed models, called the normalization model and gene model. The first estimates and removes the experiment-wise systematic errors, while the second estimates and removes the gene specific errors. The residual that remains is the log-ratio estimate for each gene. In particular, the Tukey-Kramer multiple comparison of treatment effects on each gene is performed to estimate its expression level and the significance of (confidence in) that expression level. Expresso analysis is implemented for and executed on SAS (SAS/STAT version 8.2, SAS Institute Inc., Gary, NC USA).

The original model of Wolfinger, et al., [2] includes the treatment and the array as the main effects. In previous Expresso analysis, we have extended that model to experiment-appropriate models that include additional fixed and random effects. Here, the design of the two-dye oligonucleotide microarray used in Experiment 1 and Experiment 2 includes various controls strategically positioned in different blocks of the microarray. This makes it possible to estimate the random block effect in each microarray. Furthermore, the dye effect is included in the normalization model to estimate and remove the global dye bias.

For this research, we developed two Expresso models, one whose gene model assesses the gene-(plant sample) effect (the GP model) and the other whose gene model assesses the gene-genotype-treatment effect (the GOT model). The GP model is much like previous Expresso models and is applicable to both Experiment 1 and Experiment 2. However, the GOT model is specific to analyzing Experiment 2. In both experiments, we used the GP model to estimate the differences in response of individual genotypes to treatment (drought stress versus control or ozone stress versus ambient ozone). However, the GOT model was used to estimate the effects of treatment (ozone stress), aside from the effect of individual genotypes.

#### Expresso GP model

The normalization model is

*y*_*spdab *_= *μ *+ *P*_*p *_+ *D*_*d *_+ *A*_*a *_+ (*P *× *A*)_*pa *_+ *B*_*ba *_+ *r*_*spdab*_.

Each *y*_*spdab *_value is the log_2_-transformed intensity of spot *s *within the dye *d *image in block *b *of array *a*. (A spot may represent a gene, a control, or a blank.) The global mean of the *y*_*spdab *_values, over all microarrays, is *μ*. The fixed effects in the model are the plant sample effect *P*_*p*_, where *p *indexes the various distinct plant samples from which mRNA was obtained, and the dye effect *D*_*d*_, where *d *has two values for the two dyes. The random effects in the model are the array effect *A*_*a*_, where *a *indexes the microarrays, the interaction effect (*P *× *A*)_*pa *_of plant sample *p *with microarray *a*, and the block effect *B*_*ba*_, where *b *identifies the block within microarray *a*. The model residual is *r*_*spdab*_. This differs from the normalization model in Wolfinger, et al., [2], in that it incorporates dye and block effects. It is a refinement of the Expresso normalization model in Watkinson, et al., [18], in that it has no printing pin effect, which is specific to the analysis in the earlier paper, and includes the block effect.

The second stage of the analysis uses the residual values *r*_*spdab *_computed in the first stage to estimate the interaction between an individual gene *g *and each plant sample *p *at a significance level ≤ α = 0.05. Index *g *is added to the residual values *r*_*spdab *_resulting to *r*_*gspdab*_. The value of *g *is determined using the mapping of *s *index values to *g *index values. The gene model is

*r*_*gspdab *_= *G*_*g *_+ (*G *× *P*)_*gp *_+ (*G *× *D*)_*gd *_+ (*G *× *A*)_*ga *_+ λ_*gspdab*_.

Here, *g *is a spot that represents a gene (not a blank or control) within the dye *d *image in block *b *of array *a*. The value *G*_*g *_is the mean of residual values for all spots that represent gene *g *in all images. The interactions (*G *× *P*)_*gp *_of gene *g *with plant sample *p *and of (*G *× *D*)_*gd *_of gene *g *with dye *d *are the fixed effects. The interaction (*G *× *A*)_*ga *_of gene *g *with mi-croarray a is a random effect. The λ_*gspdab *_values are stochastic errors. This differs from the gene model in Wolfinger, et al., [2], in that it incorporates interactions between gene and dye and between gene and array. It refines the Expresso gene model in Watkinson, et al., [18] to include the interaction between gene and array.

The estimate of the expression level of each gene in each treatment comparison is done by computing the pair-wise least square mean differences of gene-treatment effects. The Tukey-Kramer multiple comparison of gene-(plant sample) effects on each gene is made to estimate the *p *values associated with each calculated expression level. If there are ρ plant samples, then there are (ρ2)
 MathType@MTEF@5@5@+=feaafiart1ev1aaatCvAUfKttLearuWrP9MDH5MBPbIqV92AaeXatLxBI9gBaebbnrfifHhDYfgasaacH8akY=wiFfYdH8Gipec8Eeeu0xXdbba9frFj0=OqFfea0dXdd9vqai=hGuQ8kuc9pgc9s8qqaq=dirpe0xb9q8qiLsFr0=vr0=vr0dc8meaabaqaciaacaGaaeqabaqabeGadaaakeaadaqadaqaauaabeqaceaaaeaaiiGacqWFbpGCaeaacqaIYaGmaaaacaGLOaGaayzkaaaaaa@30FB@ possible pairwise comparisons. If we index the plant samples from 1 to ρ, then the null hypothesis for gene *g *and comparison *i*, *j*, where 1 ≤ *i *<*j *≤ ρ, is

*H*_o_: (*G *× *P*)_*gi *_= (*G *× *P*)_*gi*_.

The difference (*G *× *P*)_*gi *_- (*G *× *P*)_*gj *_is the estimate of the log_2_(fold change) of gene *g *in the experimental comparison *P*_*i *_versus *P*_*j*_. The analysis also yields a *p*-value for the statistical confidence in each difference.

The above GP model was used to analyze both Experiment 1 and Experiment 2. In both experiments, there are 48 blocks per array. In Experiment 1, there are four arrays and four plant samples, namely, WT-control, WT-stressed, antiPLD-control, and antiPLD-stressed. In Experiment 2, there are 12 arrays and eight plant samples, namely, Col-0-test, Col-0-control, Cvi-0-test, Cvi-0-control, WS-test, WS-control, Th-test, and Th-control.

#### Expresso GOT model

We wanted to estimate the gene-treatment effects separately from gene-genotype-treatment interaction effects using just one model. To do this, we designed the gene-genotype-treatment model, an alternative set of log-linear ANOVA mixed models, for the elevated CO_2 _experiment where we unfold the genotype information from the plant sample factor in the GP model. This resulted in a normalization model that includes the genotype effect (*O*_*o*_) with 4 levels (Col-0, Cvi-0, WS, and Th) and basic treatment effect (*T*_*t*_) with 2 levels (test and control). The random array (*A*_*a*_) effect however needs to be removed from the model since it confounds the genotype effect.

The normalization model is

*y*_*sotdab *_= *μ *+ *O*_*o *_+ *T*_T _+ *D*_*d *_+ (*O *× *T*)_*ot *_+ *B*_*ba *_+ *r*_*sotdab*_.

Each *y*_*sotdab *_value is the log_2_-transformed intensity of spot *s *for genotype *o *and treatment *t *within the dye *d *image in block *b *of array *a*. We have that *μ *is as in the GP model. The fixed effects in the model are the genotype effect *O*_*o*_, where *o *indexes the genotype (organism), the treatment effect *T*_*t*_, where *t *is the treatment, and the dye effect *D*_*d*_, where *d *has two values for the two dyes. The random effects in the model are the interaction effect (*O *× *T*)_*ot *_of genotype *o *with microarray *a*, and the block effect *B*_*ba*_, where *b *identifies the block within microarray *a*. The model residual is *r*_*sotdab*_. This differs from the normalization model in Wolfinger, et al., [2], in that it incorporates genotype (organism), dye, and block effects. It is a refinement of the Expresso normalization model in Watkinson, et al., [18], in that it has no printing pin effect, and includes the genotype and block effects.

The second stage of the analysis uses the residual values *r*_*sotdab *_computed in the first stage to estimate the interaction among an individual gene *g*, each genotype *o*, and each treatment *t*, at a significance level ≤ α = 0.05. Index *g *is added to the residual values *r*_*sotdab *_resulting to *r*_*gsotdab*_. The value of *g *is determined using the mapping of *s *index values to *g *index values. The gene model is

*r*_*gsotdab *_= *G*_*g *_+ (*G *× *O*)_*go *_+ (*G *× *T*)_*gt *_+ (*G *× *O *× *T*)_*got *_+ (*G *× *D*)_*gd *_+ λ_*gsotdab*_

Here, *G*_*g *_are as for the GP model. The interactions (*G *× *O*)_*go *_of gene *g *with genotype *o*, (*G *× *T*)_*gt *_of gene *g *with treatment *t*, (*G *× *O *× *T*)_*got *_of gene *g *with genotype *o *and treatment *t*, and (*G *× *D*)_*gd *_of gene *g *with dye *d *are fixed effects. The λ_*gsotdab *_values are stochastic errors. This differs from the gene model in Wolfinger, et al., [2], in that it incorporates interactions between gene and genotype, between gene and genotype with treatment, and between gene and array. It refines the Expresso gene model in Watkinson, et al., [18] to include interactions between gene and genotype and between gene and genotype with treatment.

We do pairwise comparisons as in the GP model, but there are two plausible classes of null hypotheses to test within the GOT gene model. If *τ *is the number of treatments, then the class 1 null hypothesis for gene *g *and comparison *i*,*j*, where 1 ≤ *i *<*j *≤ *τ*, is

*H*_1_: (*G *× *T*)_*gi *_= (*G *× *T*)_*gj*_.

The difference (*G *× *T*)_*gi *_- (*G *× *T*)_*gj *_is the estimate of the log_2_(fold change) of gene *g *in the *T*_*i *_versus *T*_*j*_comparison. This particular comparison looks for gene-treatment effects that are independent of genotype.

We can still estimate the expression level of each gene with respect to a specific genotype level by computing the pair-wise least square differences of gene-genotype-treatment interaction effects. The class 1 null hypothesis for gene *g *and comparison *i*, *j*, where 1 ≤ *i *<*j *≤ *τ*, is

*H*_2_: (*G *× *O *× *T*)_*goi *_= (*G *× *O *× *T*)_*goj*_.

The difference (*G *× *O *× *T*)_*goi *_- (*G *× *O *× *T*)_*goj *_is the estimate of the log_2 _(fold change) of gene *g *of a specific genotype *o *for the *T*_*i *_versus *T*_*j *_comparison. The analysis also yields a *p*-value for the statistical confidence in each difference.

The GOT model applies to Experiment 2 in a straightforward way. There are 12 arrays, two treatments, and four genotypes, namely, Col-0, Cvi-0, WS, and Th.

### TM4 microarray software suite

The TM4 microarray software suite consists of several components freely available from the TM4 web site [24]. In this research, we employed these components: ExpressConverter, Microarray Data Analysis Software (MIDAS), and Microarray Experiment Viewer (MEV). ExpressConverter converts microarray data from various data formats, such as the GenePix Results (GPR) format, to the MEV format, which is used by MIDAS and MEV. MEV format includes only integrated intensity values (IIV), which is the kind of intensity values expected of all TM4 components.

### MIDAS data normalization methods and filters

Low intensity and saturated spots are marked by quantitation programs. These spots are filtered out from the data before doing any further normalization or statistical analysis. Data normalization methods proceed from the assumption that only a relatively small proportion of the genes change significantly in expression level between the two hybridized mRNA samples. This assumption is reasonable for these data sets since the hybridizations and subsequent analysis address nearly all *Arabidopsis thaliana *genes. The MIDAS component of TM4 provides a number of data normalization methods and filters and supports applying them in a pipelined fashion [13,14].

#### Total intensity normalization

While our assumption implies that the average measured intensities of the two channels of a cDNA or oligonucleotide microarray should be almost the same, these averages are often significantly different, due to differences in the inherent fluorescence of the two dyes. The total intensity normalization step in TM4 is a straightforward means to eliminate this global dye bias. For each spot *i*, where 1 ≤ *i *≤ *n*, let *R*_*i *_and *G*_*i *_be the measured intensities of the spot in the two channels. The normalized intensity data for spot *i *is G′i
 MathType@MTEF@5@5@+=feaafiart1ev1aaatCvAUfKttLearuWrP9MDH5MBPbIqV92AaeXatLxBI9gBaebbnrfifHhDYfgasaacH8akY=wiFfYdH8Gipec8Eeeu0xXdbba9frFj0=OqFfea0dXdd9vqai=hGuQ8kuc9pgc9s8qqaq=dirpe0xb9q8qiLsFr0=vr0=vr0dc8meaabaqaciaacaGaaeqabaqabeGadaaakeaacuWGhbWrgaqbamaaBaaaleaacqWGPbqAaeqaaaaa@2F56@ = κ*G*_*i *_and R′i
 MathType@MTEF@5@5@+=feaafiart1ev1aaatCvAUfKttLearuWrP9MDH5MBPbIqV92AaeXatLxBI9gBaebbnrfifHhDYfgasaacH8akY=wiFfYdH8Gipec8Eeeu0xXdbba9frFj0=OqFfea0dXdd9vqai=hGuQ8kuc9pgc9s8qqaq=dirpe0xb9q8qiLsFr0=vr0=vr0dc8meaabaqaciaacaGaaeqabaqabeGadaaakeaacuWGsbGugaqbamaaBaaaleaacqWGPbqAaeqaaaaa@2F6C@ = *R*_*i*_, where κ is the normalization factor κ=(∑i=1nRi)/(∑i=1nGi)
 MathType@MTEF@5@5@+=feaafiart1ev1aaatCvAUfKttLearuWrP9MDH5MBPbIqV92AaeXatLxBI9gBaebbnrfifHhDYfgasaacH8akY=wiFfYdH8Gipec8Eeeu0xXdbba9frFj0=OqFfea0dXdd9vqai=hGuQ8kuc9pgc9s8qqaq=dirpe0xb9q8qiLsFr0=vr0=vr0dc8meaabaqaciaacaGaaeqabaqabeGadaaakeaaiiGacqWF6oWAcqGH9aqpcqGGOaakdaaeWaqaaiabdkfasnaaBaaaleaacqWGPbqAaeqaaaqaaiabdMgaPjabg2da9iabigdaXaqaaiabd6gaUbqdcqGHris5aOGaeiykaKIaei4la8IaeiikaGYaaabmaeaacqWGhbWrdaWgaaWcbaGaemyAaKgabeaakiabcMcaPaWcbaGaemyAaKMaeyypa0JaeGymaedabaGaemOBa4ganiabggHiLdaaaa@4680@ Quackenbush [14] discusses this normalization in the context of sev-eral variations that are possible to address differing channel intensities.

#### Lowess normalization

Beyond the global dye bias, there is dye bias that is dependent on the measured spot intensities [25,26]. TM4 constructs a scatter plot, called an RI-plot, of the points (*x*_*i*_, *y*_*i*_), where 1 ≤ *i *≤ *n*, given by *x*_*i *_= log_10_(*R*_*i*_*G*_*i*_) and *y*_*i *_= log_2_(*R*_*i*_/*G*_*i*_). Under our assumption, the RI-plot should be very nearly symmetric with respect to the line *y *= 0. In lowess normalization, TM4 applies the lowess method of Cleveland [27] to fit a locally weighted regression curve to the RI-plot; TM4 then adjusts spot intensities to eliminate any systematic intensity-dependent bias. Additional details on correcting intensity-dependent bias is found in [14].

#### Standard deviation regularization

After total intensity and lowess normalizations eliminate dye bias on a global (per microarray) scale, TM4 employs standard deviation regularization to ensure that the per-block variances of log(*R*_*i*_/*G*_*i*_) values are the same [25,28]. Quackenbush [14] provides the formulas for this normalization step.

#### Low intensity filtering

Since the relative error in the log(*R*_*i*_/*G*_*i*_) values increases if *R*_*i *_or *G*_*i *_is close to background levels, spots with low intensities are filtered out. Quackenbush [14] provides additional details, which essentially require that both *R*_*i *_and *G*_*i *_intensities be above two standard deviations of the respective backgrounds.

#### The MIDAS pipeline

We applied a MIDAS pipeline consisting of total intensity normalization, lowess normalization, standard deviation regularization, and low intensity filtering to both microarray data sets. MIDAS default parameters were used throughout; the default low intensity filter cut-off is *R*_*i*_*G*_*i *_< 10,000.

### TM4 MEV analysis

The Multi Experiment Viewer (MEV) component of TM4 provides a number of statistical analyses and clustering algorithms to identify differentially expressed genes. We report results from the one-class *t*-test analysis applied to output of the MIDAS pipeline. This test assumes that the paired distribution of treated and control groups is normally distributed. Since the intensities measured from the same spot are correlated, we can apply the one-class *t*-test for the two-group comparison.

## Authors' contributions

AAS performed the data preprocessing, Expresso analysis of all data sets, developed the database of statistical results, and drafted the manuscript. SPM performed the drought stress experiment (experiment 1), while PL performed the elevated CO_2 _experiment (experiment 2). PL performed the qRT-PCR of 192 genes from experiment 2. WS and PL performed TM4 analysis of experiments 1 and 2 respectively. LSH supervised the data analysis. LSH, HJB, and RG conceived of the study and coordinated the work. All authors read and approved the manuscript.

## Supplementary Material

Additional File 1 **Intensity-dependent dye bias in Rl-plots**. Supplementary Figure 1 is a PDF file that contains six RI-plots that illustrate specific intensity-dependent dye bias as the microarray data is processed through the normalization steps. Click here for file

Additional File 2 Genes subjected to qRT-PCR. Supplementary Table 1 is a PDF file that contains the annotation of genes subjected to qRT-PCR and the functional categories represented. For verification of microarray results in Experiment 2, Li, *et al*., [23] performed real-time quantitative reverse-transcriptase PCR (qRT-PCR) for selected genes — 55 in Col-0; 52 in Cvi-0; 59 in WS; 26 in Th. The AT numbers of those genes, their annotation, and categorizations into biological functions are in the table. Click here for file

## Figures and Tables

**Table 1:** Number of differentially expressed genes. Numbers of identified differentially expressed genes in the GP and GOT models after preprocessing by 0 or more MIDAS pipeline steps — IIV, IIV_T_, IIV_TL_, IIV_TLS_, or IIV_TSLF_. For Experiment 1, up-expression (+) and down-expression (-) numbers are given for both WT and antiPLD. For Experiment 2, + and -1 numbers are given for all 4 genotypes separately. The ALL entries correspond to the H1 hypotheses of the GOT model (see Materials and Methods).

Genotype		IIV	IIV_T_	IIV_TL_	IIV_TLS_	IIV_TLSF_
		GP Model — Experiment 1				
		
WT	+	567	571	487	495	460
WT	-	552	553	499	499	460
antiPLD	+	442	421	440	471	431
antiPLD	-	336	346	381	363	334

		GOT Model — Experiment 2				
		
Col-0	+	227	309	242	240	270
Col-0	-	122	171	178	180	183
Cvi-0	+	106	108	82	78	71
Cvi-0	-	63	93	74	71	67
WS	+	234	346	279	269	150
WS	-	331	347	340	340	224
Th	+	238	398	350	363	361
Th	-	196	245	241	246	240
ALL	+	298	428	357	354	290
ALL	-	202	261	253	248	195

**Table 2:** Retention counts and percentages. Retention counts (RC) and retention percentages (RP) for the differentially expressed gene sets of Table 1. RC is the number of genes in a set before the MIDAS step that remain in the set after that step. RC is the number of genes in the set before the MIDAS step that remain in the set after that step. RP is the percentage of remaining genes with respect to the number of genes in the set after the MIDAS step. RC and RP are reported for the intersections IIV_∩ _IIV_T_, IIV_T _∩ IIV_TL_, IIV_TL _∩ IIV_TLS_, and IIV_TLS _∩ IIV_TLSF_, as well as intersection IIV_∩ _∩IIV_TLSF_, which corresponds to the effect of MIDAS steps from the start of the pipeline to the end. The ALL entries correspond to the H1 hypotheses of the GOT model (see Materials and Methods).

		IIV ∩		IIV_T_∩		IIV_TL_∩		IIV_TLS_∩		IIV ∩	
		IIV_T_		IIV_TL_		IIV_TLS_		IIV_TLSF_		IIV_TLSF_	
						
Genotype		RC	RP	RC	RP	RC	RP	RC	RP	RC	RP
		GP Model — Experiment 1									
		
WT	+	545	95.45	74	15.20	285	57.58	456	99.13	59	12.83
WT	-	532	96.20	55	11.02	306	61.32	459	99.78	53	11.52
antiPLD	+	403	95.72	58	13.18	278	59.02	430	99.77	46	10.67
antiPLD	-	326	94.22	45	11.81	203	55.92	330	98.80	33	9.88

		GOT Model — Experiment 2									
		
Col-0	+	224	72.49	221	91.32	219	91.25	172	63.70	159	58.89
Col-0	-	121	70.76	150	84.27	165	91.67	120	65.57	92	50.27
Cvi-0	+	81	75.00	65	79.27	71	91.25	49	69.01	36	50.70
Cvi-0	-	60	64.52	60	81.08	63	88.73	41	61.19	38	56.72
ws	+	223	64.45	230	82.44	248	92.19	108	72.00	101	67.33
ws	-	293	84.44	267	78.53	314	92.35	180	80.36	149	66.52
Th	+	236	59.30	308	88.00	330	90.91	256	70.91	201	55.68
Th	-	183	74.69	210	87.14	225	91.46	172	71.67	144	60.00
ALL	+	282	65.89	307	85.99	333	94.07	200	68.97	177	61.03
ALL	-	196	75.10	196	77.47	230	92.74	133	68.21	103	52.82

**Table 3:** Comparison of MIV and IIV. We compare MIV and IIV input data with respect to the sets of genes ultimately identified as differentially expressed. The genotype and set labelings are the same as those in Table 1. The GP model was used to analyze the unnormalized MIV and IIV data from Experiment 1. The GOT model was used to analyze the unnormalized MIV and IIV data from Experiment 2. Up-expressed and down-expressed counts are reported for both experiments and all genotypes. The count of genes in the intersections is found in column Common. For convenience, percentages of the intersection with respect to the MIV and IIV sets are tabulated in the last two columns. The ALL entries correspond to the H1 hypotheses of the GOT model (see Materials and Methods).

Genotype		MIV	IIV	Common	% of common in MIV	% of common in IIV
		GP Model — Experiment 1				
		
WT	+	725	567	501	69.10%	88.36%
WT	-	774	552	515	66.54%	93.30%
antiPLD	+	518	422	357	68.92%	84.60%
antiPLD	-	505	336	313	61.98%	93.15%

		GOT Model — Experiment 2				
		
Col-0	+	311	227	198	63.67%	87.22%
Col-0	-	190	122	110	57.89%	90.16%
Cvi-0	+	185	106	89	48.11%	83.96%
Cvi-0	-	97	63	59	60.82%	93.65%
ws	+	326	234	181	55.52%	77.35%
ws	-	482	331	273	56.64%	82.48%
Th	+	363	238	219	60.33%	92.02%
Th	-	251	196	166	66.14%	84.69%
ALL	+	448	298	249	55.58%	83.56%
ALL	-	294	202	159	54.08%	78.71%

**Table 4:** Comparison of Expresso analysis and MEV *t *test. We compare the number of genes identified as differentially expressed by Expresso analysis and the MEV *t*-test. Counts for both up-expressed and down-expressed genes, as well as all four genotypes of Experiment 2, are reported. The first three analyses — the GP model, the GOT model, and the MEV *t*-test — take the IIV_TLSF _data as input. For point of comparison, the last analysis uses the GP model on MIV input.

	GP Model		GOT Model		MEV *t*-test		GP Model	
	IIV_TLSF_		IIV_TLSF_		IIV_TLSF_		MIV	
				
Genotype	+	-	+	-	+	-	+	-
Col-0	1837	1563	270	183	2125	2007	1761	1212
Cvi-0	817	1248	71	67	478	823	669	1403
WS	2606	2002	150	224	535	388	2422	1184
Th	2016	1314	361	240	1144	827	2395	1457

Totals	7276	6127	852	714	4282	4045	7247	5256

**Table 5:** Comparison of qRT-PCR with Expresso and TM4. We compare the qRT-PCR results with identified up-expressed and down-expressed genes in Experiment 2 using Expresso and TM4. Results of qRT-PCR are available for each of the four ecotypes in the numbers *n *in the second column. Numbers of agreement or non-agreement are shown in the S, D, and F columns. The S (same) column tabulates the number of genes for which the sign of the log(fold change) for statistically significant differential expression matches the direction of change in the corresponding qRT-PCR result. The D (differing) column tabulates the number of genes for which the sign of the log(fold change) for statistically significant differential expression is in the opposite direction of the change in the corresponding qRT-PCR result. The F (filtered) column tabulates the number of genes for which there is a qRT-PCR result, but for which either the gene was filtered by MIDAS low intensity filtering or the analysis method did not assess the change in expression as statistically significant. The MEV *t*-test (first grouping) results are for the typical TM4 process, which involves IIV input data followed by the four MIDAS steps, which we denote IIV_TLSF _The GP model (second grouping) gives the same numbers and uses the same IIV_TLSF_input data, the GP model (third grouping) results use MIV input data and has no filtered genes.

		GP Model		GP Model			MEV *t*-test		
	qRT-PCR	MIV		IIV_TLSF_			IIV_TLSF_		
				
Genotype	*n*	S	D	S	D	F	S	D	F
Col-0	55	50	5	43	8	4	40	9	4
Cvi-0	52	46	6	38	9	5	36	7	9
ws	59	54	5	46	10	3	41	9	9
Th	26	23	3	21	3	2	19	3	4

Total	192	173	19	148	30	14	138	28	26
Percentage		90.1%	9.9%	77.1%	15.6%	7.3%	71.9%	14.6%	13.5%

**Table 6:** Correlation of qRT-PCR with Expresso and TM4. We calculate the correlation of qRT-PCR results from the Expresso GP model and the MEV *t*-test. For each comparison, the analytical result for each gene for which there is a qRT-PCR result for a particular genotype is assembled into a result vector. We used SAS to compute a Pearson correlation of each result vector with the corresponding vector of qRT-PCR results. The computed correlations are as reported above.

	Genotype-Specific Correlation			
	
Comparison	Col-0	Cvi-0	WS	Th
qRT-PCR versus GP model with MIV input	0.85	0.73	0.66	0.84
qRT-PCR versus GP model with IIV_TLSF _input	0.83	0.69	0.61	0.78
qRT-PCR versus MEV *t*-test with IIV_TLSF _input	0.83	0.65	0.64	0.77
