# Supplementary material for: The statistics of identifying differentially expressed genes in Expresso and TM4: a comparison
Source: BMC Bioinformatics. 2006 Apr 20;7:215. doi: 10.1186/1471-2105-7-215 (PMC1513403; doi:10.1186/1471-2105-7-215)
Supplement: Additional File 1 — Intensity-dependent dye bias in Rl-plots. Supplementary Figure 1 is a PDF file that contains six RI-plots that illustrate specific intensity-dependent dye bias as the microarray data is processed through the normalization steps. [file 1471-2105-7-215-S1.pdf]

RI Plots

Additional File 1. Supplement to BMC Bioinformatics paper *The statistics of identifying differentially expressed genes in Expresso and TM4: a comparison,* by Allan A. Sioson, Shrinivasrao P. Mane, Pinghua Li, Wei Sha, Lenwood S. Heath, Hans J. Bohnert, and Ruth Grene, 2006.

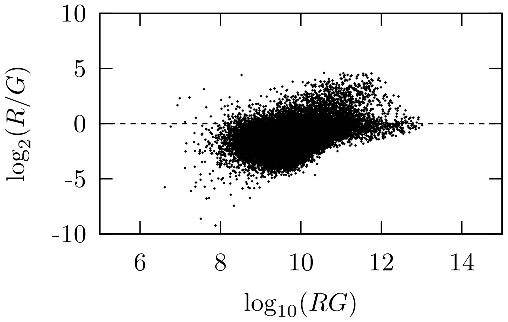

(a) Original integrated intensity values

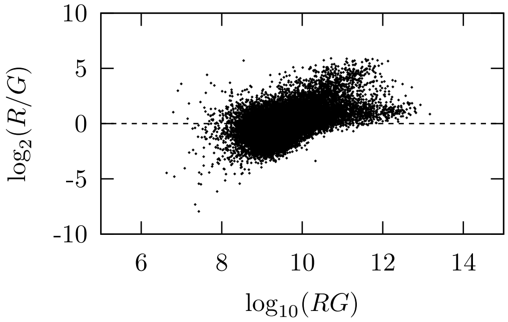

(b) Expresso normalization applied to (a)

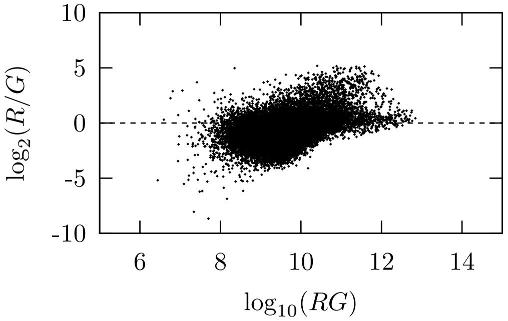

(c) Total intensity normalization applied to (a)

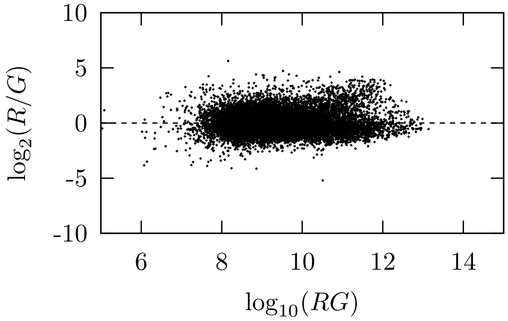

(d) Lowess normalization applied to (c)

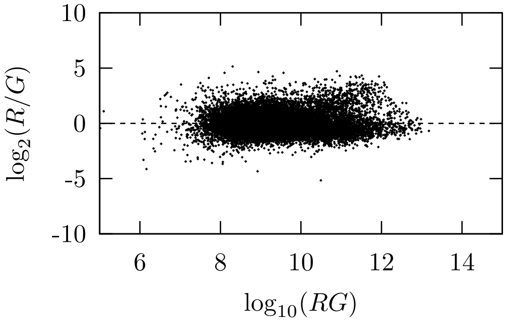

(e) Standard deviation regularization applied to (d)

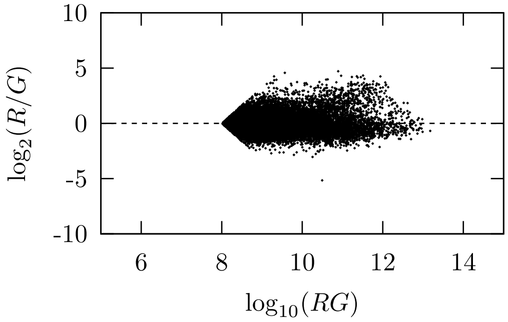

(f) Low intensity filtering applied to (e)

Supplementary Figure 1: RI-plots illustrating specific intensity-dependent dye bias as microarray data is processed. (a) The original data (IIV) for the Experiment 1, WT plant samples, and second replicate microarray. The intensity-dependent dye bias is apparent in the RI-plot of this

unnormalized data set. (b) Expresso normalization ($\mathrm{IIV_E}$, where E signifies Expresso normalization) applied to IIV. This shifts the plot up so that the points are evenly distributed above and below the x-axis, though it does not correct the intensity-dependent bias. (c) MIDAS total intensity normalization ($\mathrm{IIV_T}$) applied to IIV. This makes little difference in the RI-plot and certainly does not correct the intensity-dependent bias. (d) MIDAS lowess normalization ($\mathrm{IIV_{TL}}$) applied to $\mathrm{IIV_T}$. Visually, this appears to remove some of the intensity-dependent bias, though the result does not appear as good as that in Figure 2 of Quackenbush [14]. (e) MIDAS standard deviation regularization ($\mathrm{IIV_{TLS}}$) applied to $\mathrm{IIV_{TL}}$. There is little effect. (f) MIDAS low intensity filtering ($\mathrm{IIV_{TLSF}}$) applied to $\mathrm{IIV_{TLS}}$. This eliminates spots with RG values below 10,000. Again, there is little effect.
