# Supplementary material for: The statistics of identifying differentially expressed genes in Expresso and TM4: a comparison
Source: BMC Bioinformatics. 2006 Apr 20;7:215. doi: 10.1186/1471-2105-7-215 (PMC1513403; doi:10.1186/1471-2105-7-215)
Supplement: Additional File 2 — Genes subjected to qRT-PCR. Supplementary Table 1 is a PDF file that contains the annotation of genes subjected to qRT-PCR and the functional categories represented. For verification of microarray results in Experiment 2, Li, et al., [23] performed real-time quantitative reverse-transcriptase PCR (qRT-PCR) for selected genes — 55 in Col-0; 52 in Cvi-0; 59 in WS; 26 in Th. The AT numbers of those genes, their annotation, and categorizations into biological functions are in the table. [file 1471-2105-7-215-S2.pdf]

Genes Subjected to qRT-PCR

Additional File 2. Supplement to BMC Bioinformatics paper *The statistics of identifying differentially expressed genes in Expresso and TM4: a comparison,* by Allan A. Sioson, Shrinivasrao P. Mane, Pinghua Li, Wei Sha, Lenwood S. Heath, Hans J. Bohnert, and Ruth Grene, 2006.

Supplementary Table 1: Annotation of genes subjected to qRT-PCR and the functional categories represented. For verification of microarray results in Experiment 2, Li et al. [23] performed real-time quantitative reverse-transcriptase PCR (qRT-PCR) for selected genes — 55 in Col-0; 52 in Cvi-0; 59 in WS; 26 in Th.

Annotation of qRT-PCR Genes	
**Amino Acid Metabolism**	
AT5GG5010	asparagine synthetase 2 (ASN2)
**Carbon Metabolism**	
AT1G09960	sucrose transporter / sucrose-proton symporter (SUT4)
AT1G12240	beta-fructosidase (BFRUCT4) / beta-fructofuranosidase / invertase, vacuolar
AT1G22710	sucrose transporter / sucrose-proton symporter (SUC2)
AT1G35580	beta-fructofuranosidase / invertase / saccharase / beta-fructosidase
AT1G43670	fructose-1,6-bisphosphatase / D-fructose-1,6-bisphosphate 1-phosphohydrolase
AT1G58180	carbonic anhydrase family protein / carbonate dehydratase family protein
AT1G73110	ribulose bisphosphate carboxylase/oxygenase activase / RuBisCO activase
AT1G73370	sucrose synthase / sucrose-UDP glucosyltransferase
AT2G21590	ADPglucose pyrophosphorylase Large subunit 3, APL3
AT2G31390	pfkB-type carbohydrate kinase family protein
AT2G35840	sucrose-phosphatase 1 (SPP1)
AT3G01500	carbonic anhydrase 1, chloroplast / carbonate dehydratase 1 (CA1)
AT3G06500	beta-fructofuranosidase / invertase / saccharase / beta-fructosidase
AT3G29360	UDP-glucose 6-dehydrogenase
AT3G43190	sucrose synthase / sucrose-UDP glucosyltransferase
AT3G46970	Glucan phosphorylase (cytosolic), PHS2
AT3G52340	sucrose-phosphatase 3b (SPP3b)
AT4G03050	encodes a 2-oxoglutarate-dependent dioxygenase
AT4G09020	isoamylase / starch debranching enzyme

Continued on next page

Annotation of qRT-PCR Genes	
AT4G26620	sucrase-related
AT4G37870	phosphoenolpyruvate carboxykinase (ATP) / PEP carboxykinase / PEPCK
AT4G39210	ADPglucose pyrophosphorylase Large subunit 4, APL4
AT5G11110	SPS5a, sucrose-phosphate synthase -like protein
AT5G22510	beta-fructofuranosidase / invertase / saccharase
AT5G43450	1-aminocyclopropane-1-carboxylate oxidase
AT5G51830	pfkB-type carbohydrate kinase family protein
AT5G55900	sucrase-related

Hormones	
AT1G14920	gibberellin response modulator (GAI) (RGA2) / gibberellin-responsive modulator
AT2G19590	1-aminocyclopropane-1-carboxylate oxidase / ACC oxidase

Lipid Metabolism	
AT5G01220	UDP-sulfoquinovose:DAG sulfoquinovosyltransferase / sulfolipid synthase (SQD2)

Nucleic Acids	
AT2G30470	ABI3/VP1 family regulatory protein

Secondary Metabolism	
AT3G51240	naringenin 3-dioxygenase / flavanone 3-hydroxylase (F3H)
AT5G16450	dimethylmenaquinone methyltransferase family protein

Signaling	
AT2G19860	hexokinase 2 (HXK2)
AT4G14110	COP9 signalosome subunit / CSN subunit (CSN8), CSN8, FUS7
AT4G26080	ABI1 protein phosphatase ABI1
AT4G29130	hexokinase 1 (HXK1)
AT5G02290	serine/threonine-specific protein kinase NAK
AT5G57050	ABI 2 protein phosphatase 2C

Continued on next page

Annotation of qRT-PCR Genes	
**Stress**	
AT1G70290	TPS8, trehalose-6-phosphate synthase, putative
**Transport**	
AT1G44100	amino acid permease 5
AT1G61800	glucose-6-phosphate/phosphate translocator
AT1G67940	ABC transporter family protein
AT1G71890	sucrose transporter / sucrose-proton symporter (SUC5)
AT2G02860	sucrose transporter / sucrose-proton symporter (SUC3)
AT5G54800	glucose-6-phosphate/phosphate translocator
**Uncategorized**	
AT2G34810	putative berberine bridge enzyme
AT4G15610	integral membrane family protein
AT5G57685	expressed protein

Conclusion of table
